# Uterine choriocarcinoma associated with ovarian cysts in a *Cavia porcellus*: a clinical case

**DOI:** 10.3389/fvets.2026.1818533

**Published:** 2026-05-20

**Authors:** Alessandro Vetere, Martina Gavezzoli, Viola Bigazzi, Melania Di Pentima, Federico Armando, Martina Fumeo, Benedetta Passeri, Francesco Di Ianni

**Affiliations:** 1 Veterinary Teaching Hospital, Department of Veterinary Medical Sciences, University of Parma, Parma, Italy; 2 Department of Veterinary Science, Pathology Unit, University of Parma, Parma, Italy

**Keywords:** *Cavia porcellus*, choriocarcinoma, hematuria, varian cysts, ovariohysterectomy

## Abstract

A 3. 5-year-old female guinea pig was presented for suspected hematuria. Hematology, serum biochemistry, urinalysis, and fecal examination were within normal limits; however, diagnostic imaging revealed multiple ovarian cysts and a uterine mass without evidence of metastasis. The animal underwent an ovariohysterectomy to remove the mass but died 6 h after anesthetic recovery. Histopathological examination identified a uterine adenoma with focal choriocarcinoma, associated with polycystic ovarian disease. To the authors' knowledge, this represents only the second reported case of uterine choriocarcinoma in the guinea pig. To the authors' knowledge, this is the first reported case of uterine choriocarcinoma in a guinea pig documented with a complete clinical, surgical, diagnostic imaging, histopathological, and necropsy evaluation.

## Introduction

Guinea pigs (*Cavia porcellus*) are spontaneous ovulators with an estrous cycle of approximately 15–17 days (1). It has been reported that this cycle can be monitored through vaginal cytology, which shows variations in epithelial and leukocyte populations throughout the cycle (1).

Understanding the estrous cycle is essential for interpreting reproductive pathologies and planning clinical interventions, as these are commonly observed in intact, older females, with ovarian cysts representing one of the most frequently diagnosed conditions (2, 3).

These cysts are typically classified into two main types: non-functional serous cysts, which are often asymptomatic, and hormonally active follicular cysts, which, although non-neoplastic, may induce clinical signs such as bilateral symmetrical flank alopecia, abdominal distension, and infertility (4, 5). Diagnosis is generally based on compatible clinical signs and is confirmed through ultrasonographic evaluation. The occurrence of ovarian cysts does not appear to be influenced by the reproductive history of the animal (5), suggesting that age and ovarian physiology may play a more important role. The hormonal content of ovarian cysts has been measured, with estradiol (~61.9 pg/ml) and progesterone (~2.7 ng/ml) detected in the cyst fluid; however, a clear correlation with systemic plasma levels has not been established, and the effect on uterine hormonal stimulation remains unclear (6).

Hormonally active cysts may promote endometrial proliferation and predispose affected females to secondary uterine disorders, most commonly cystic endometrial hyperplasia and endometritis, which can progress to bacterial infections and pyometra, reported in both breeding and non-breeding animals (2, 7). Less frequently, these pathological changes may be associated with uterine and ovarian neoplasms, including leiomyomas, granulosa cell tumors, leiomyosarcomas, endometrial adenomas, adenocarcinomas, anaplastic tumors of unknown origin, and choriocarcinomas (2, 8). Clinical signs of uterine and ovarian tumors often overlap with other reproductive disorders and include hemorrhagic vaginal discharge, abdominal distension, palpable abdominal masses, and abdominal pain (7).

A retrospective histological study of 37 uterine lesions in guinea pigs revealed that 35% were neoplastic, while the remaining lesions consisted of cystic endometrial hyperplasia (66%), endometrial hemorrhage (12.5%), pyometra (8.3%), and mucometra (3.7%) (8). Among the 13 neoplastic lesions, leiomyomas (46%) and adenomas (23%) were the most frequent, followed by leiomyosarcomas (7.6%), anaplastic tumors of unknown origin (15.3%), and choriocarcinomas (7.6%) (8). The treatment of choice for these reproductive pathologies, whether cystic, infectious, or neoplastic, is generally surgical, with ovariohysterectomy being recommended; hormonal therapy may be considered only in select cases of hormonally responsive ovarian cysts (4, 7). Definitive tumor classification relies on histopathological examination following surgical removal of the reproductive organs (8).

## Case description

A 3.5-year-old intact female *Cavia porcellus* was presented to the veterinary hospital for evaluation of blood spots observed on the bedding. On clinical examination, the patient had a body condition score of 7/9 and was normothermic. Cardiac auscultation revealed no abnormalities, and abdominal palpation identified a soft, non-painful abdomen. No blood was detected in the perineal region.

Complete blood count, serum biochemistry, and urinalysis showed no significant abnormalities, and fecal examination was negative for parasites.

Based on the initial clinical presentation, differential diagnoses included urolithiasis, urinary tract infection, pyometra, endometritis, neoplastic disease of the urogenital tract, and hemorrhagic diarrhea. As no definitive diagnosis could be established, further diagnostic investigations were performed with owner consent.

Abdominal ultrasound revealed multiple round anechoic structures in both ovaries, consistent with ovarian cysts. The body of the uterus was moderately distended with anechoic fluid, and cranially, a 4 × 4.3 cm heterogeneous, hypoechoic, and vascularized ovoid mass was observed, suggestive of a neoplasm. Whole-body radiographs (lateral and ventrodorsal views) showed no evidence of pulmonary metastases. A contrast-enhanced total body CT scan confirmed the presence of a 4.8 x 4.2 x 2.6 cm heterogeneously enhancing mass in the left uterine horn, with hypoattenuating, non-enhancing internal areas likely consistent with necrosis, bilateral multiple ovarian cysts, and the absence of metastases (Supplementary Video 1).

Given these findings, an ovariohysterectomy was performed. Premedication consisted of methadone (Semfortan^®^, Dechra Veterinary Products, Northwich, UK) (0.5 mg/kg), dexmedetomidine (Dexdomitor^®^, Orion Corporation, Espoo, Finland) (50 μg/kg), and ketamine (Nimatek^®^, Dechra Veterinary Products S.r.l., Turin, Italy) as part of a multimodal anesthetic protocol, which allowed a dose-sparing approach for all drugs while maintaining adequate anesthetic depth. Induction and maintenance were performed with isoflurane (IsoFlo^®^, Zoetis Inc., Parsippany-Troy Hills, NJ, USA) (2.5%) via facemask.

**Figure 1:**
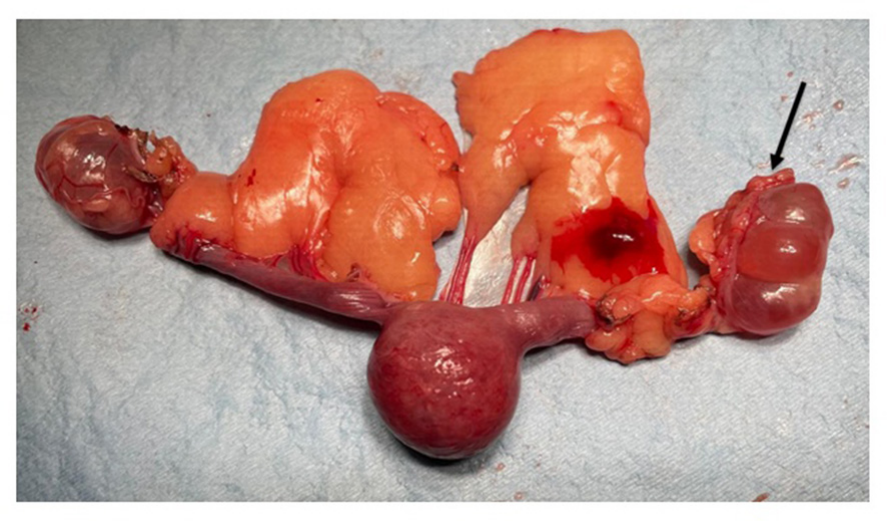
Gross pathology of the genital tract. There is a bilateral, multiloculated cystic *rete ovarii* (black arrow) and a solid, nodular, hemorrhagic mass that involves the uterine body.

The procedure was completed successfully, with no intraoperative complications observed. Vital parameters, including heart rate, respiratory parameters, and body temperature, remained stable and within physiological limits throughout the procedure. Gross examination of the excised uterus revealed a solid, highly vascularized mass measuring 3.5 × 4 cm, with areas of macroscopic necrosis (Figure 1).

**Figure 2:**
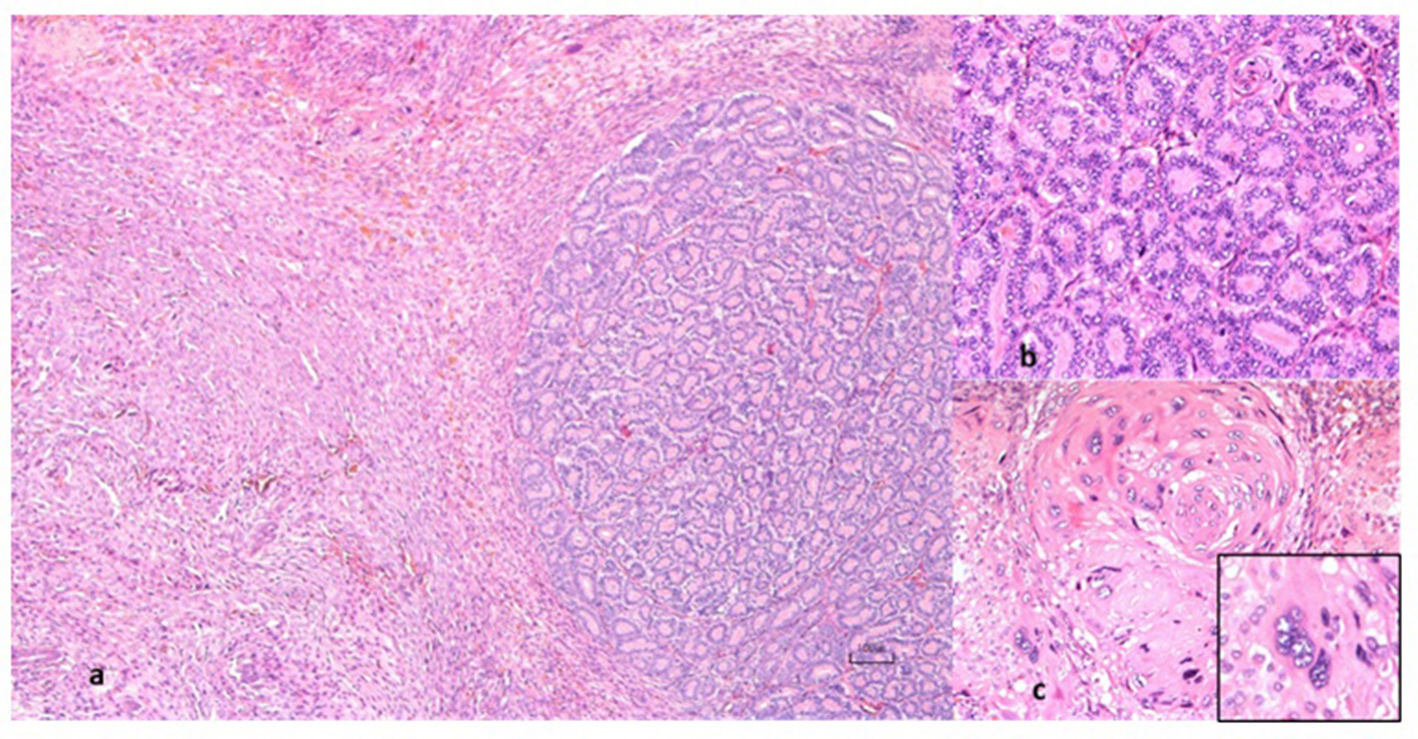
Histopathology of the uterine nodular lesion. **(a)** there is a nodular, densely cellular, epithelial cell proliferation arising from the uterine mucosa. **(b)** well differentiated tumoral area with neoplastic cells forming tubules supported by a scant fibrovascular stroma. **(c)** poorly differentiated tumoral area with pleomorphic neoplastic cells forming solid foci. The high magnification insert shows a cell with variable cytoplasm, five large, central vesicular nuclei (syncytiotrophoblast).

Unfortunately, the patient died approximately 6 h postoperatively, exhibiting neurological signs and acute respiratory distress. Histopathological examination of the uterine lesion was performed. The uterine mucosal, submucosal and muscular layers are circumferentially expanded and multifocally infiltrated by an epithelial, densely cellular, neoplastic proliferation that is well demarcated and not encapsulated. Neoplastic cells are mainly arranged in tubular structures supported by a scant fibrovascular stroma that occasionally becomes highly cellular and dense in collagen (desmoplastic response). Cells are cuboidal, with well-defined cell borders, intermediate nucleus/cytoplasmic ratio and a moderate amount of homogeneous to granular eosinophilic cytoplasm. Nuclei are round, parabasal, with vesicular chromatin and 1–2, small, basophilic evident nucleoli. Anisocytosis and anisokaryosis are moderate, there is occasional karyomegaly and mitoses are 0–5 in 2.37mm^2^. Within this population there are multifocal areas showing a decreased differentiation with a more pleomorphic morphology ranging from polygonal to spindloid. Other areas show groups of moderate numbers of cells, forming rather solid areas and characterized by abundant to moderate homogeneous cytoplasm, 1–5 large, central vesicular nuclei (syncytiotrophoblasts). Altogether these morphological features were highly suggestive of a uterine carcinoma with areas of choriocarcinomatous differentiation (Figure 2).

Necropsy revealed no additional significant findings.

## Discussion

The present case aligns with the literature regarding the frequent occurrence of ovarian cysts and their association with secondary uterine lesions, while highlighting the exceptionally rare finding of choriocarcinomatous differentiation (9). To provide a clearer context for this common condition, which is only rarely associated with neoplastic entities, a structured comparison of the main histopathological cyst types, their imaging features, and size ranges is summarized in Table 1 (10–12). The coexistence of polycystic ovaries and a uterine neoplasm mirrors patterns observed in retrospective studies, in which neoplastic lesions frequently occur alongside cystic or inflammatory uterine conditions (8).

**Table 1:** Comparative overview of the main types of ovarian cysts in *Cavia porcellus*.

Features	Rete ovarii cysts (serous)	Follicular cysts
Origin	Rete ovarii (embryonic remnant)	Graafian follicles (anovulatory)
US features	Anechoic, thin-walled, often multilocular	Anechoic, thin-walled, often unilocular
Hormonal activity	Historically non-functional (but see estradiol/progesterone levels)	Steroidogenic (Estrogens/Androgens)
Clinical signs	Asymptomatic or mass effect (pain >10.4 mm)	Bilateral alopecia, clitoral hypertrophy

Uterine tumors with choriocarcinomatous differentiation are extremely rare in guinea pigs, with only one previously reported case described in the literature (8). Notably, in that case, it was not specified whether the diagnosis was made ante-mortem or post-mortem. In contrast, the present case represents one of the first reports in which the diagnosis was established clinically, supported by imaging and confirmed by histopathology following ovariohysterectomy, rather than being discovered solely at necropsy.

Clinically, the presentation of subtle blood spots on the bedding underscores that reproductive neoplasms in guinea pigs may be asymptomatic or show non-specific signs, with normal hematology, biochemistry, and urinalysis. Imaging modalities, including ultrasonography and CT, effectively identified ovarian cysts and the uterine mass while excluding metastases. However, imaging alone cannot predict histologic complexity or malignant potential, highlighting the indispensable role of histopathology.

Ovariohysterectomy remains the treatment of choice for cystic, infectious, and neoplastic reproductive pathologies (4, 7). Despite an uneventful surgical procedure, the patient died postoperatively, likely due to anesthetic or perioperative complications, illustrating the challenges of anesthesia and postoperative management in small exotic mammals, which are more complex due to their reduced size and the limited species-specific data available. No intraoperative bleeding was observed, and postoperative hemorrhage was excluded based on necropsy findings.

In other species, including dogs, cats, and humans, choriocarcinomas are treated surgically if localized, with systemic chemotherapy reserved for metastatic or unresectable disease. Common regimens in humans include methotrexate, actinomycin D, or combination protocols. To our knowledge, no chemotherapeutic interventions have been reported for uterine choriocarcinoma in guinea pigs, likely due to their rarity and the sensitivity of the species to cytotoxic drugs (13, 14).

The authors recognize a main limitation of this study. Due to the lack of cross-reactive antibodies and the lack of specific trophoblastic markers, we could not perform immunohistochemistry on the examined samples. However, due to the peculiar morphological features of trophoblastic cells, we feel that the final diagnosis and the scientific content of the entire case report is adequate. This case emphasizes the importance of including reproductive tract pathologies in the differential diagnosis of intact older females presenting with subtle clinical signs and supports regular monitoring. Early ovariohysterectomy may be considered as a preventive measure to reduce the risk of reproductive neoplasia.

## Data Availability

The raw data supporting the conclusions of this article will be made available by the authors, without undue reservation.

## Supplementary material

Supplementary Video Contrast-enhanced total body CT scan.
